# Electrochemistry in sensing of molecular interactions of proteins and their behavior in an electric field

**DOI:** 10.1007/s00604-023-05999-2

**Published:** 2023-10-17

**Authors:** Jan Vacek, Martina Zatloukalová, Vlastimil Dorčák, Michal Cifra, Zdeněk Futera, Veronika Ostatná

**Affiliations:** 1 Department of Medical Chemistry and Biochemistry, Faculty of Medicine and Dentistry, Palacky University, Hnevotinska 3, 77515 Olomouc, Czech Republic; 2 Institute of Photonics and Electronics of the Czech Academy of Sciences, Chaberska 1014/57, 18200 Prague, Czech Republic; 3 Faculty of Science, University of South Bohemia, Branisovska 1760, 37005 Ceske Budejovice, Czech Republic; 4 Institute of Biophysics, The Czech Academy of Sciences, v.v.i., Kralovopolska 135, 61200 Brno, Czech Republic

**Keywords:** Protein, Peptide, Electrode, Sensor, Microdevice

## Abstract

Electrochemical methods can be used not only for the sensitive analysis of proteins but also for deeper research into their structure, transport functions (transfer of electrons and protons), and sensing their interactions with soft and solid surfaces. Last but not least, electrochemical tools are useful for investigating the effect of an electric field on protein structure, the direct application of electrochemical methods for controlling protein function, or the micromanipulation of supramolecular protein structures. There are many experimental arrangements (modalities), from the classic configuration that works with an electrochemical cell to miniaturized electrochemical sensors and microchip platforms. The support of computational chemistry methods which appropriately complement the interpretation framework of experimental results is also important. This text describes recent directions in electrochemical methods for the determination of proteins and briefly summarizes available methodologies for the selective labeling of proteins using redox-active probes. Attention is also paid to the theoretical aspects of electron transport and the effect of an external electric field on the structure of selected proteins. Instead of providing a comprehensive overview, we aim to highlight areas of interest that have not been summarized recently, but, at the same time, represent current trends in the field.

**Graphical abstract:**

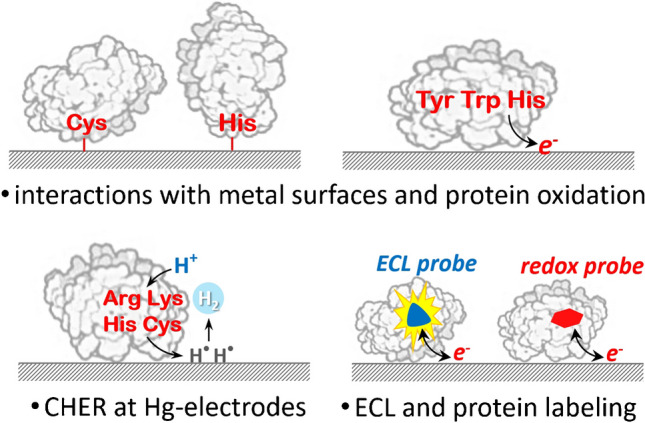

## Introduction

Proteins are structural, functional, and regulatory elements of cells and tissues. Current knowledge about proteins is closely connected with progress in structural biology research and development and the determination of the structure of a number of proteins [1], as well as in analytical instrumentations and methodologies. Interest in the electrochemical analysis of proteins was initiated only 6 years after J. Heyrovský’s invention of polarography [2] by discovering a new phenomenon manifested by the ability of proteins to catalyze hydrogen evolution at a dropping mercury electrode (DME). Circumstances leading to this significant discovery (in the absence or presence of cobalt ions), along with early polarographic investigations of proteins, are described in the literature [3, 4].

Later, the DME was gradually replaced with gold, silver, platinum, and graphite electrodes modified with various adsorbates to study a relatively small group of conjugated proteins, usually containing non-proteinaceous redox-active metal centers (prosthetic groups or cofactors) that provided fast reversible redox electrode processes. Great interest in this branch of protein electrochemistry (also called protein film voltammetry or protein film electrochemistry) was induced by a very important finding, showing that appreciable electron transport between the electrode and protein redox-active centers, which are not accessible to the electrode surface, can be achieved using an effective electron transfer mediator [5, 6]. Mostly, proteins containing heme, iron-sulfur, or copper redox centers are investigated, and methodologies for their attachment onto a variety of conducting surfaces and assemblies of them that are useful for probing biological redox processes have been recently reviewed [7–9].

**Fig. 1:**
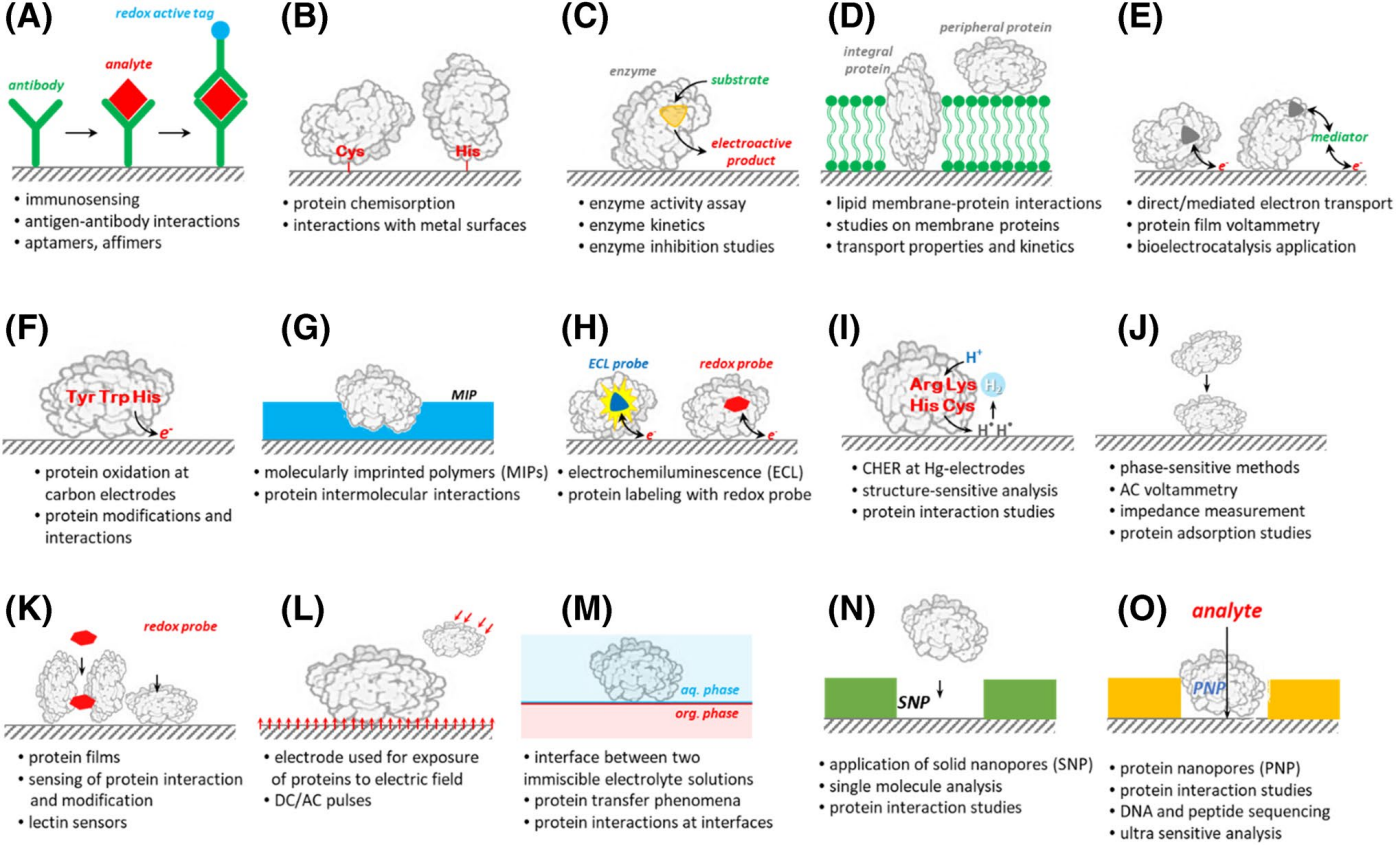
Overview of protein electrochemical approaches. (**A**) Protein sensors based on biorecognition elements [12, 13]. (**B**) Protein chemisorption *via* metal-binding *aa* (Cys and His) residues, with consequent metal complex reduction [23]. (**C**) Enzyme electrode-generated redox-active reaction product [24]. (**D**) Protein/lipid layer/electrode architecture and derived approaches [11]. (**E**) Protein film voltammetry and bioelectrocatalysis applications [5, 6]. (**F**) Oxidation of protein *aa* residues, mainly Tyr and Trp [25]. (**G**) Proteins and MIP technology [26]. (**H**) ECL methods [12] and labeling with redox-active probes (more details in the “Electroactive redox labels in protein sensing” section). (**I**) Catalytic hydrogen evolution reaction of proteins [27]. (**J**) Protein surface adsorption, desorption, and reorientation processes [28]. (**K**) Electrochemical impedance analysis [29]. (**L**) Electric field effects both in bulk and at the interface, as discussed in the “Electro-manipulation of protein structure and function” section. (**M**) Protein behavior at the electrified interface of two immiscible liquids [30]. (**N**) Nanopores as a selective barrier for proteins and peptides and (**O**) protein nanopore working as a selective barrier (or environment) for various analytes [20–22]

The isolation and structural elucidation of key membrane proteins, both transporters and receptors, gave a new impulse to electrochemical studies of proteins connected with the development of various biomimetic membranes or simple detergent and lipid layers [10, 11]. Furthermore, due to advances in the construction of various electrochemical (bio)sensors and proteomic approaches [12, 13], labeling proteins with redox-active probes has been established, and electrochemiluminescence has been utilized for protein analysis [13]. Today’s trends are also directed toward the electrochemical sensing of proteins at the atomic level and taking advantage of computational tools to study protein interactions with electrode surfaces and electron transfer phenomena in general [14]. Finally, it is worth mentioning that close attention has to be paid to the adsorption of proteins onto electrode surfaces, as well as to the structural and functional changes that may occur in them when exposed to an electric field, to avoid misinterpretation of the results obtained, particularly in investigations of protein structures and interactions with other bio(macro)molecules or substances [15–18]. As for future trends in single-molecule analysis and sequencing, the application of proteins as nanopores, and nanopore technologies in general, are also very promising [19–22]. A schematic overview of general electrochemical approaches useful for protein studies is shown in Fig. 1.

This text does not aim to provide a comprehensive overview, but instead to point out individual trends, especially in research on the electroactivity of non-conjugated and membrane proteins. Furthermore, we aim to describe the effects that occur after the exposure of proteins to an electric field, and advanced computational tools are also highlighted. In addition, we summarize the basic strategies in protein microanalysis and labeling (or electrochemically promoted labeling) with redox-active probes. Particular attention is paid to areas not reviewed in this form in recent years.

For further study, we recommend the following comprehensive reviews on proteomics and glycomics [18], membrane proteins [11], and on the electrochemical research of peptides [15]. Historical aspects in this regard have also been recently reported [31].

## Intrinsic electroactivity of proteins

Over the last few decades, significant progress in the electrochemical analysis of proteins has been made using constant-current chronopotentiometric stripping (CPS) in combination with mercury-containing electrodes (reviewed in [17, 18, 25, 27]). Under conditions close to physiological, proteins containing Arg, Lys, Cys, or His amino acid (*aa*) residues [32–34] produce a well-developed peak (so-called peak H) due to the catalytic hydrogen evolution reaction (CHER) [18, 35]. Methods based on CPS peak H can be applied for the label-free reagentless structure-sensitive analysis of practically any protein, since proteins that do not contain any of these residues, if any, are extremely rare. An important condition for obtaining protein peak H is the accessibility of the catalytically active *aa* residues for the electrode process. In a native folded protein, *aa* residues buried in the interior of the molecule and/or those located far from the electrode surface can remain catalytically silent. On the other hand, they may become involved in CHER after the protein’s denaturation [25, 36]. Even the first works utilizing CPS peak H demonstrated the possibility of studying local and global changes in protein structures [25, 36] due to the ability of surface-attached proteins to retain their folded structures close to the potential of zero charge, but undergo time-dependent denaturation/unfolding at negatively charged surfaces [17, 37, 38]. The denaturation of surface-attached proteins can be minimized by adjusting the duration of the protein exposure to the electric field to milliseconds [39], as well as other experimental conditions, such as solution temperature [37] and ionic strength [40]. The high sensitivity of the CPS peak H to structural changes in proteins can be utilized not only for monitoring protein denaturation [41–43], oligomerization and aggregation [44, 45], posttranslational modifications [46–48], oxidative damage [49, 50], and single-*aa* replacements [51, 52] but also for investigating protein interactions with DNA [53–55], peptides [56], and other proteins [57–60]. CPS peak H appeared to be particularly useful for analyzing water-soluble and membrane proteins [11, 61–65]. All these analyses are based on utilizing the different accessibilities of electroactive residues, which is influenced by the adsorption and/or structural stability of the given protein or its complex. Peak H appears at highly negative potentials close to –1.7 V (*vs.* Ag|AgCl|3M KCl). At such negative electrode potentials, an extremely high electric field (10^9^ V/m) [66] can affect the electric double-layer with adsorbed biomolecules. Moreover, it can cause DNA melting, protein denaturation, complex disaggregation, etc. [18].

**Fig. 2:**
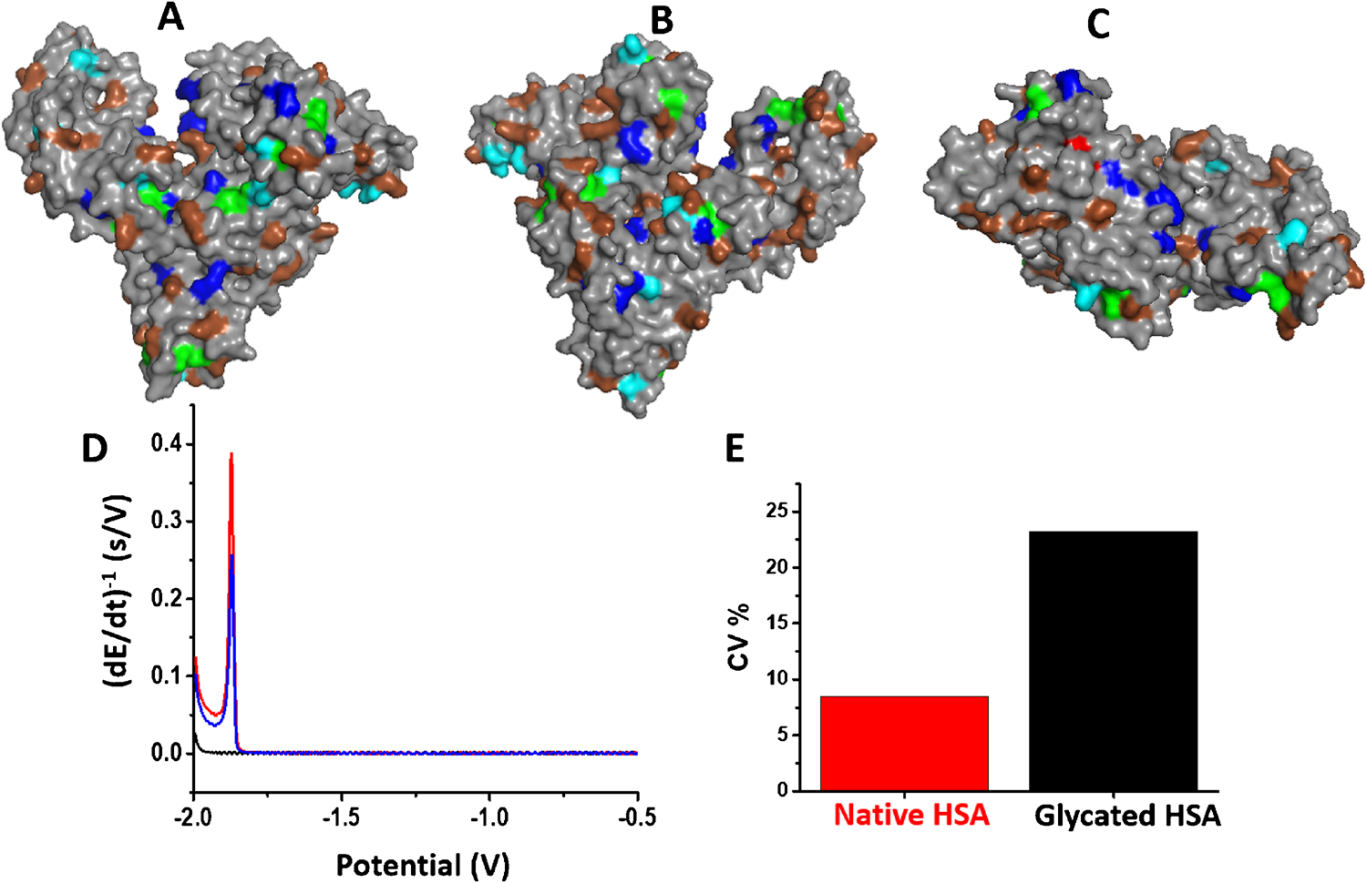
Surface models of HSA (1BJ5) with electrocatalytically active *aa* residues highlighted: Lys-brown, His-cyan, Arg-blue, Cys-green, and Cys34-red. The left (**A**) and right (**B**) images are mutually rotated by 180° along the vertical axis for each panel. (**C**) Free Cys34 (red highlighted) is visible from the top image. (**D**) CPS records (peaks H) of native (red line) and artificially glycated with methylglyoxal (blue line) HSA isolated from the blood serum of a healthy subject; see ref. [67]. The black line indicates blank – supporting electrolyte. (**E**) Coefficient of variation (CV) for CPS response of native albumin samples and after their artificial glycation

**Fig. 3:**
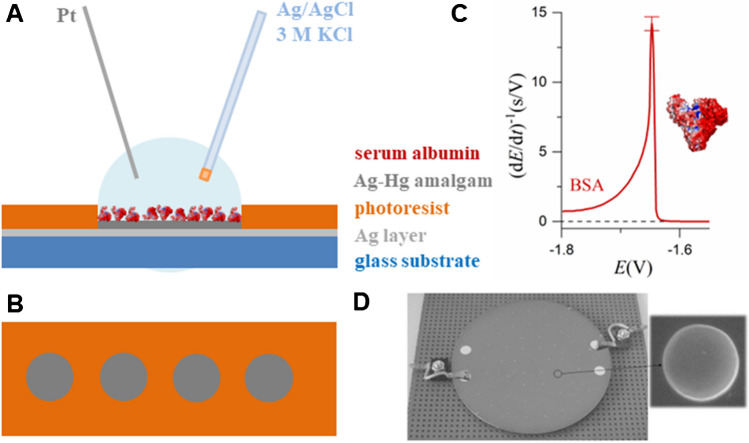
Cross-section (**A**) and upper view (**B**) of electrode wells. (**A**) Schematic representation of CPS analysis of bovine serum albumin (BSA) in the well of an array. (**C**) Chronopotentiogram of 5 μM BSA (red, solid line) adsorbed from 20 μl drop for accumulation time of 60 s from background electrolyte, McIlvaine buffer, pH 7 (black, dashed line). (**D**) Final electrode array on insulating pad. Reprinted with permission from [70]. Copyright 2010 American Chemical Society

CPS peak H was also utilized to study proteins in complex media [53] as well as to analyze clinical samples, such as human serum albumin (HSA) samples isolated from blood serum [67]. The surface distribution of *aa* residues active in CHER in the HSA molecule is shown in Fig. 2A–C. HSA isolated from healthy volunteers gave a CPS peak H which decreased after modification of the sample with methylglyoxal (MGO); see Fig. 2D. MGO is a reactive metabolite that is able to modify the same *aa* residues that are involved in the CHER. Based on the results of this study, the coefficient of variation for the native albumin samples was estimated to be 8.5%, while that for the inter-individual binding capacity variations, evaluated using the artificial-glycation/carbonylation approach, was 23.2 % (Fig. 2E). Recently, interactions of HSA with fatty acids and their nitro-derivatives were also investigated using CPS peak H [68, 69]. In addition to hanging mercury drop electrode (HMDE), CPS analysis in combination with an Ag-amalgam electrode microdevice can be effectively applied for CHER monitoring, which was demonstrated in bovine serum albumin sensing; see Fig. 3 [70].

In addition to proteins, also some peptides, oligo- and poly-nucleotides, and oligo- and polysaccharides have been found to be catalytically active in the hydrogen evolution reaction at mercury-containing electrodes (reviewed in the literature [15, 17, 18, 27]). Nevertheless, despite some attempts to better understand CHER at the fundamental level [32–35, 71–74], methodologies utilizing CHER are still mostly based on empirical findings, and further research is necessary to exploit the full potential of this electrocatalytic phenomenon in the research of various bio(macro)molecules and their mutual interactions.

Besides the electrocatalytic reduction occurring at mercury-containing electrodes, the oxidation reactions of proteins at carbon electrodes have also been found to be useful for their label-free analysis [18, 25, 75–77]. Even though some *aa’s*, namely Trp, Tyr, Cys, His, and Met, are oxidizable at carbon electrodes [18, 78–80], it is predominantly Tyr and Trp residues that have yielded well-developed oxidation peak/s with proteins [78]. Peptides and small proteins yielded separated peaks of Tyr and Trp residues [75, 81]. However, larger proteins, in most cases, only produced a single peak. The well-developed Tyr and/or Trp peak/s, in contrast to the poor or absent peaks of Met, His, and Cys residues in proteins, could be due to the stronger interactions of Tyr and Trp residues with the electrode surface than those of other electroactive residues, as a theoretical study showed [82]. A well-developed His peak was observed for oncoprotein AGR2 modified with a His-tag [80], since the six linked His residues on its terminus are more accessible for oxidation than those buried inside the protein structure. *Aa* residues of proteins are more exposed after protein denaturation. Denaturation of the AGR2 protein led to the appearance of a negligible His peak and an increase in the Tyr and Trp peak [80]. Severalfold higher Tyr and Trp peaks for denatured forms than those for native ones were also reported for other proteins [78, 83–85]. Oxidation of the Tyr and/or Trp residues was also found to be useful for studying the oligomerization and aggregation of alpha-synuclein and amyloid peptides [86–88]. Similarly, the posttranslational modification of peptide and protein Tyr and Trp residues, such as phosphorylation [89] and nitration [90], as well as oxidative damage [50] and ligand binding [69, 91], had an impact on the oxidation responses of Trp or Tyr residues.

## Electroactive redox labels in protein sensing

**Fig. 4:**
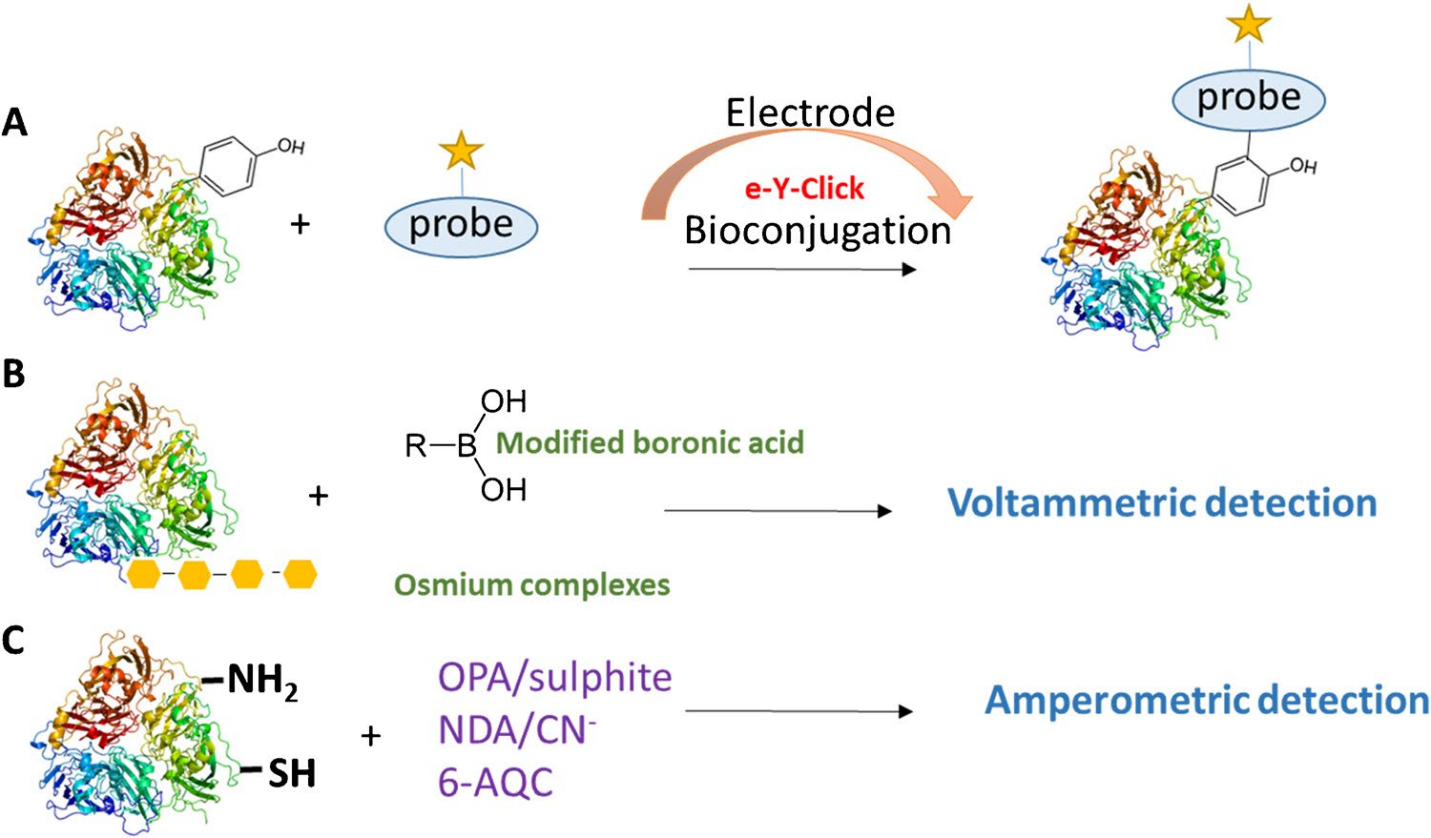
Schematic representation of protein/peptide labeling. (**A**) Labeling based on electrochemically promoted Tyr-click-chemistry, (**B**) electrochemical sensing of glycoprotein using boronic acid or osmium complexes, and (**C**) derivatization approaches for electrochemical detection; see refs. [76, 95–99]. For other details, including abbreviations, see the “Electroactive redox labels in protein sensing” section

Protein labeling is generally performed *via* Lys residues with *N*-hydroxysuccinimide-activated esters, sulfonyl chlorides, or iso(thio)cyanates. Another option is Cys labeling by Michael reaction or reactions that target electron-rich Tyr or Trp residues [92, 93]. The electrochemically promoted Tyr-modification of peptides and proteins with labeled urazoles was studied at the low potential of +0.36 V (*vs*. Ag|AgCl|sat. KCl). Under these conditions, the urazole anchors could be activated without oxidizing the sensitive *aa* residues in the protein. Protocols were successfully performed in the electrosynthesis of peptides and proteins, such as oxytocin, angiotensin, BSA, and epratuzumab. An electrochemically promoted labeling approach was also developed for Tyr-containing proteins with phenothiazine derivatives [94]. The electro-oxidation of phenothiazine produces a nitrogen radical cation, which reacts with the *ortho* position of the Tyr phenol. Two proteins, which contain Tyr on the protein surface, insulin, and myoglobin, were modified with phenothiazine [94]. Similar to the electrochemically promoted Tyr-click reaction, a bioconjugation reaction for selective Trp labeling in peptides and proteins has been developed (Fig. 4A) [100].

Boronic acid functionalized compounds have been utilized for biosensing glycoproteins. A schematic representation of the interaction of glycoprotein with modified boronic acid is shown in Fig. 4B. Boronic acids interact with 1,2- or 1,3-diols of saccharide to create five/six-membered cyclic complexes and also interact with Lewis bases to form boronate anions [101]. An amperometric sensor was constructed for monitoring fructosyl valine, the glycosylated part of hemoglobin, based on soluble ferrocenylboronic acid [102]. The glycosylated part of hemoglobin was investigated at the carbon electrode *via* a ferrocene moiety. An electrochemical method for glycoprotein detection based on 4-mercaptophenylboronic acid (MBA)/biotin-modified gold nanoparticles (AuNPs) was used for the study of recombinant human erythropoietin (rHuEPO) as a model protein. In more detail, rHuEPO was first captured by an electrode covered with anti-rHuEPO aptamer and then derivatized with MBA-biotin-AuNPs. The MBA-biotin-AuNPs interact with streptavidin-conjugated alkaline phosphatase to produce electroactive *p*-aminophenol [7]. Electrochemical biosensors based on MBA-capped AuNPs were also used for monitoring a prostate-specific antigen and avidin. A sub-picomolar limit of detection of avidin/prostate-specific antigen was achieved [103].

There have been several studies focused on utilizing osmium complexes for the electrochemical analysis of peptides and proteins. A complex composed of osmium tetraoxide and 2,2´-bipyridine was used for the labeling and electrochemical detection of Trp residues of salmon and human luteinizing hormone [104], avidin, streptavidin, and lysozyme [105]. Osmium(VI) complexes (ligands: 2,2′-bipyridine and *N*,*N*,*N*′,*N*′-tetramethylethylenediamine) were also used for labeling the sugar part of glycoproteins with an electrochemical detection endpoint. This approach was applied to determine RNase B and avidin, with the limit of detection (LOD) ranging between 25 and 50 nM. Electrochemical signals were monitored at a pyrolytic graphite electrode by adsorptive transfer stripping square-wave voltammetry [106, 107].

The most commonly used detectors coupled to separation techniques in proteomics are mass spectrometry and laser-induced fluorescence detection. An alternative to the above-mentioned detectors is electrochemical detection, especially amperometric and pulse amperometric detection [95]. The direct detection of a redox-active *aa* on carbon electrodes or the utilization of metal-based solid electrodes is limited by the LOD [76]. The most commonly used derivatization agents in terms of *aa’s* and peptides are *o*-phthaldialdehyde (OPA) and naphatalene-2,3-dicarboxyaldehyde (NDA) in the presence of a nucleophile (sulfur derivatives or CN^−^), reviewed in the literature [95]. Other agents utilized in the derivatization of *aa’s* are 6-aminoquinolyl-*N*-hydroxysuccinimidyl carbamate (6-AQC) [96, 97] and *p*-nitrophenol-2,5-dihydroxyphenylacetate bis-tetrahydropyranyl ether [98, 99]. The protein interaction with the most common derivatization agents is demonstrated in Fig. 4C.

A peptide-1 probe (RNRCKGTDVQAW) was designed as an electroactive label of daunomycin for ovalbumin protein recognition. The peak current of the daunomycin moiety decreased with increasing concentration of ovalbumin due to the interaction between ovalbumin and the electroactive peptide probe. Differential pulse voltammograms of daunomycin and labeled peptides in the presence or absence of ovalbumin were obtained using a glassy carbon electrode. According to this protocol, the concentrations of ovalbumin in the egg whites were measured with a detection limit at the 10^−11^ M level.

An electrochemical sensor was also developed [108] for monitoring the following protein kinases: sarcoma-related kinase, extracellular signal-regulated kinase 1, and cyclin A-dependent kinase 2*.* The approach is based on the ability of kinases to transfer a redox-labeled phosphoryl group, the specific substrate for the protein kinase, to surface-bound peptides. Voltammetric and electrochemical impedance spectroscopic detection was enabled due to 5′-γ-ferrocenoyl-ATP, a co-substrate for peptide phosphorylation. The labeling strategies are schematically summarized in Fig. 4.

## Nano/micro materials in protein electrochemistry: double-surface technique

In the last decade, there has been a significant increase in published studies in which the authors use a variety of nano- and micromaterial technologies for the study and sensitive electrochemical analysis of proteins. Very often, these are applications of carbon or metal nanoparticles, which are used to decorate the surfaces of the electrodes, often complex multi-layer or multi-component modifications [109]. These systems are effectively applied to increase the working (active) surface of the sensor, improve the sensitivity/selectivity of the determination, or increase the electron transfer rate between the protein and the electrode, e.g., in the development of enzyme electrodes or biofuel cells [110]. On the other hand, a certain disadvantage of these procedures could be the poor reproducibility of the preparation of such complex electrode architectures. In fact, for fundamental research, it is usually best to use an unmodified (bare) electrode with a well-defined and reproducible surface. In addition, the preparation of complicated (e.g., sandwich configuration) structures on the surfaces of electrodes goes against the main added value of electrochemical determinations, which is the simplicity (“elegance”) of the experimental setup, possibly even the minimal financial demands of performing such analysis or research; see a recent historical review [111].

One of the other possibilities where we can use nano/micro technologies in the electrochemistry of biomacromolecules is the concept of the double-surface technique (DST) [112, 113]. This is based on the application of microparticles or a selected nano/micro material (“first surface”) for the manipulation or purification of the investigated proteins before their adsorption onto the electrode detection surface (“second surface”) and subsequent electrochemical analysis. After the release of the protein from the first surface, the adsorptive transfer (AdT) technique [114] can be used. This method allows the protein to be adsorbed onto the surface of the electrode from microliter volumes, and after washing the electrode, the biopolymer-modified electrode is inserted into an electrochemical cell containing an already pure supporting electrolyte. For DST purposes, magnetic nano- or micromaterials can be beneficial. Originally, these approaches were applied to the study of DNA interactions and hybridization. In this particular case, magnetic beads (magnetoseparation) were used [115, 116]. DNA was bound (anchored) to their surface, most often *via* a terminal oligo(A) sequence or a biotinylated terminus to the oligo(T) chain or (strept)avidin immobilized on the surface of magnetic beads, e.g., ref. [117]. The immobilized DNA (but also RNA) can be easily purified (washing step) and further incubated. The washing is based on the repetitive magnetic attraction of the beads to the wall of the plastic microtube and the consequent resuspension step, which allows multiple purification cycles [115]. Subsequently, the target biopolymer is released from the magnetic carrier and dissolved in a buffer or medium that is fully compatible and optimized for analysis.

**Fig. 5:**
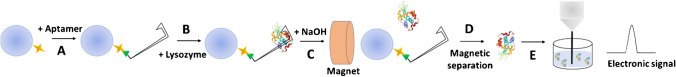
Electrochemical sensing of aptamer-protein interactions. (**A**) Attachment of biotinylated anti-lysozyme aptamer to streptavidin-modified magnetic microbeads. (**B**) Binding of target protein. (**C**) Alkaline-induced release of captured protein. (**D**) Magnetic separation step. (**E**) CPS detection of released protein in connection with adsorptive accumulation [120]

This technique is applicable to the research of protein-DNA interactions [118, 119] and can also be used for the electrochemical analysis of proteins, both labeled and unlabeled, according to the approaches mentioned above in the text. In such cases, proteins can be bound to magnetic particles using antibodies or aptamers (Fig. 5) [120].

## Electro-manipulation of protein structure and function

**Fig. 6:**
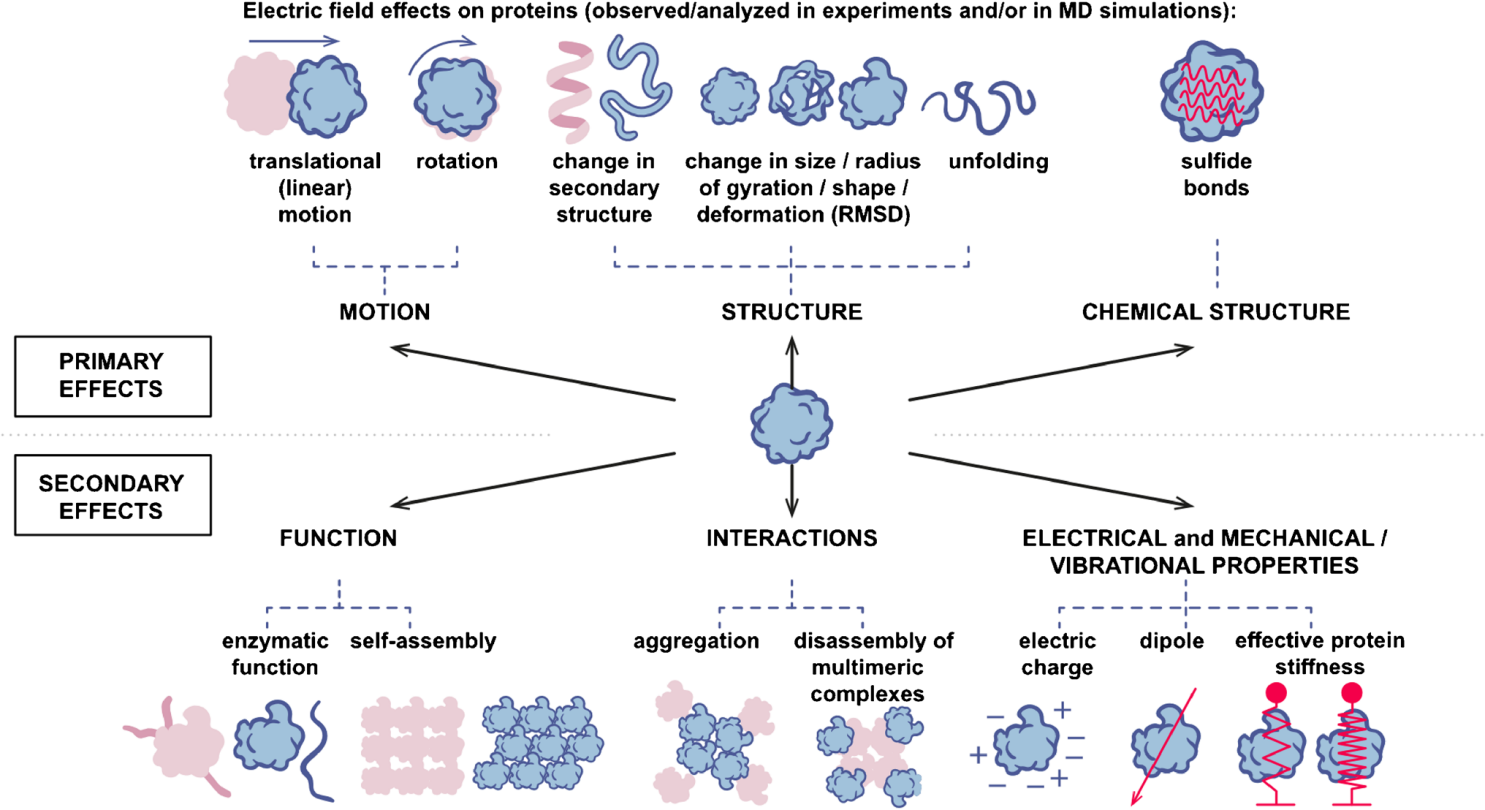
Electric field exerts effects on protein *via* force on electrically charged groups. The effects can be primary in character, such as electrophoretic linear motion, rotation, change in secondary structure, and protein shape. These primary effects then translate to secondary effects on protein electrostatic and mechanical properties, multivalent interaction between the proteins, as well as protein function. For more details, see the main text

An electric field (EF) acts as a direct force on charged groups in the proteins, and an EF can also act on a protein indirectly through its action on the charges of the surrounding ions and solvent. The presence of ions and (polarizable) solvent also decreases the effective EF strength by Coulombic screening [121]. The most trivial effect is the net translation (electrophoretic) force on a protein (see Fig. 6) used in a variety of separation and detection techniques. An EF, even an intrinsic protein EF, also naturally acts on electrostatic interactions (including Coulomb interactions) in the protein [122] and on protein-solvent interactions. The electric double-layer around the protein, a simplified picture of the complex charge distribution at the interface of the protein and solvent [123], can be potentially also perturbed by an external EF affecting the balance of the forces of the protein. The charge distribution on the protein itself forms an effective dipole, on which the EF acts as a torque, leading to the rotation of the protein [124, 125]. Electric forces can also cause overall deformation [126] of the protein, leading to a change in the secondary structure and, provided the electric force is high enough, ultimately to unfolding [127–129]. All the above-mentioned effects of EF on protein structure can lead to a plethora of functional effects.

Electric fields in the form of short (nanosecond-microsecond) intense pulses (pulsed electric field, PEF) are of particular interest for the electro-manipulation of proteins for several reasons. First, very high electric fields (>units and tens of MV/m) are strong enough to affect the protein structure [126]. Second, intense electric pulses of nanosecond-microsecond duration can only carry a small amount of energy, so they cause little to no heating. Appropriate guidance by theories and models is needed to rationally guide the formulation of hypotheses for experiments. Computational molecular dynamics (MD) simulation is such a modeling tool, furthermore with the ultimate spatial and temporal resolution, so far unmatched by any experimental technique. Hence, MD simulations enable exploration of the effects of an EF on molecules and proteins at the atomistic level and with time resolution down to femtoseconds [130, 131]. Using MD simulations, it has been demonstrated that an intense EF can rotate the protein, affect (i) the protein’s secondary and tertiary structure [132–134], (ii) the radius of the gyration [135–137], (iii) dipole moment [138, 139], and ultimately lead to unfolding [140, 141]. At the level of peptide and protein ensembles, the EF causes disaggregation and the detachment of subunits from multimeric protein complexes [142, 143].

Here, we highlight effects which most commonly appear in the literature analyzing the effect of an intense EF on proteins *in silico*. One of the direct effects of EF on proteins is the rotation of the protein by a torque exerted by the EF. The dipole moment of a protein arises from the charge distribution in the protein, and the effects of EFs on protein dipole moments have been extensively studied using MD simulations [130, 143–146]. Myoglobin polarization under pulsed/static EFs exhibited a fast transition with high-intensity EFs and an increase in dipole moment with lower-intensity EFs, despite minimal impact on the protein’s structure or geometry [140]. Tubulin proteins, with a high charge and dipole moment, exhibited polarization-induced changes in shape and orientation under EFs, influencing binding sites and potential applications in protein-drug interactions and ion channels [133]. Recent work has highlighted the non-linear effects of high-intensity continuous-wave EFs and emphasized the importance of operating within a “weak field condition intensity range” in MD simulations to avoid significant non-linear and saturation effects [147, 148]. Manipulating protein orientation through their dipole moment using EFs also has promising implications in the X-ray imaging of single molecules, allowing structure determination with smaller sets of diffraction data [149]. Simulations demonstrated an “orientation window” of field strengths in which proteins maintained intact structures, while longer exposure times shifted the window toward lower fields, suggesting “orientation before destruction” [125, 149].

Other substantial effects of a strong EF on proteins are the change in the protein’s secondary structure and unfolding. For example, extensive MD simulations on myoglobin showed that both static and nanosecond pulsed EFs disrupt about 70% of its α-helical secondary structure [140]. However, EF intensities below 100 MV/m have no observable impact on the secondary structure or geometry of myoglobin [140]. Insulin’s response to EFs varied based on the type and intensity, with 500 MV/m causing more significant disruption than static fields of the same intensity [150]. Studies on hen egg white lysozyme revealed denaturation under oscillating EFs, while high field strengths induced similar unfolding pathways [144, 145]. MD simulations of the SOD1 enzyme showed that 100 MV/m had no effect on secondary structures, 500 MV/m caused partial denaturation, and 700 MV/m led to complete unfolding [151]. The unfolding of ubiquitin protein using static EFs exhibited an intensity-dependent speed, with medium and high strengths inducing rapid unfolding, where deliberate unfolding using EFs provided valuable insights into protein stability [141]. There is also growing experimental evidence for a variety of these simulation predictions. For example, it was demonstrated that an intense EF affects the secondary structure of BSA [152], whey proteins [153], and lysozyme [154] using circular dichroism spectroscopy.

At the level of larger protein structures and polymers, it was shown, for example, that an intense EF can significantly affect the microtubule (MT) lattice. It was found that a nanosecond-scale intense electric field can induce a longitudinal opening of the cylindrical shell of the MT lattice, modifying the structure of the MT. This effect is field strength- and temperature-dependent and occurs on the cathode side [143]. MD simulations suggest that a high EF strength (at least tens or even a few hundreds of MV/m) is required to affect protein structures.

**Fig. 7:**
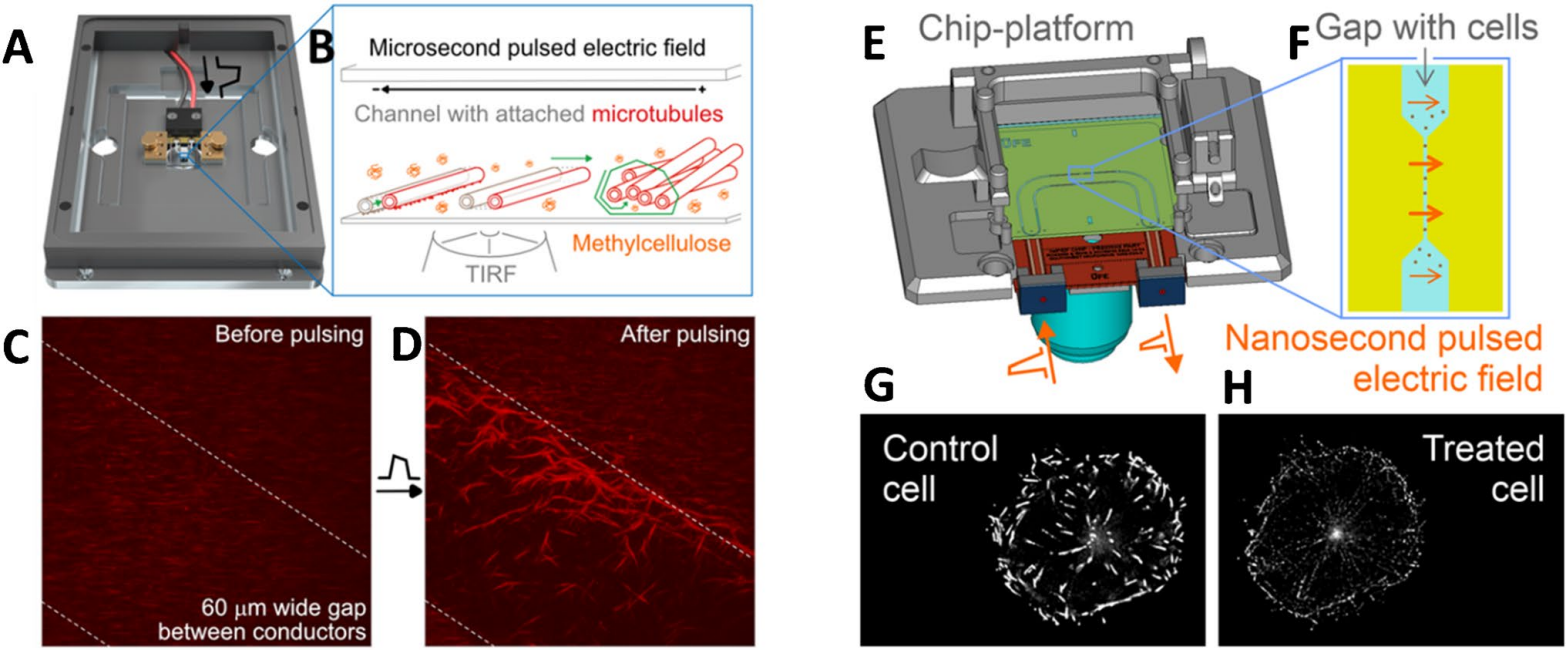
(**A**) Total-internal reflectance fluorescence (TIRF) microscopy platform with chip (adapted from [155]) (**B**) demonstrates the capability of the chip – detachment of antibody-bound microtubules from a surface and their translocation and concentration. (**C**, **D**) TIRF microscopy images before and after the application of 100× 5 μs, 2.5 MV/m s electric pulses fired at 10 Hz frequency, microtubules are red-labeled fibers. (**E**) Coplanar waveguide–based chip platform on structured illumination microscope (SIM), adapted from [156]. (**F**) Cells are placed on a tapered gap between the ground and the central conductor. (**G**, **H**) SIM images of control cell and cell treated with 4000× 10 ns, 6 MV/m electric pulses fired at 100 Hz

It is often difficult to achieve such values of field strength while being able to observe the behavior of proteins at the same time. To address this challenge, there is an ongoing development of new technological platforms (Fig. 7) which integrate planar electromagnetic chips into advanced light microscopy and spectroscopy systems [157–159]. These chips enable the controlled delivery of intense short electric pulses to protein samples, while the microscopes make it possible to observe the response of proteins to the EF *in situ*, in real time, and in a biologically relevant chemical environment. For instance, the delivery of 6 MV/m 11 ns pulses to biosamples on such a chip integrated into a structured illumination microscope has been recently demonstrated [156]. In that study, it was shown that these electric pulses can remodel the cellular microtubule network in rat basophilic leukemia (RBL) cells. In a follow-up work, it was revealed that the pulsed EF (PEF) also exerts similar effects on the microtubule network in human osteosarcoma (U2OS) cells as well as retinal pigment (RPE1) cells [160]. Furthermore, several works showed that μs and ns electric pulses affect the microtubule cytoskeleton in a variety of cells; see more in a recent review [161]. These effects of intense PEF delivered in short pulses on the microtubule network in cells are very promising for potential therapeutic applications, but the inevitable side effects of ns PEF on cellular complexity and the cell membrane obscure the mechanism of action. In short, the effects observed on microtubules in cells could be an effect of downstream signaling due to the primary action of PEF on the membrane (causing electroporation) or on membrane voltage-gated ion channels.

Therefore, there is an ongoing effort to understand the effects of PEF on well-defined reconstituted systems, such as giant unilamellar vesicles [162], vesicles with actin [163], or isolated protein structures [164–166]. For example, a study was conducted to examine the direct impact of PEF on tubulin [109]. There, nanosecond PEF was applied to isolated unpolymerized tubulin, and it changed the tubulin’s self-assembly capability. The change was reversible or irreversible, depending on the pulse parameters.

We foresee that the combination of chips enabling pulsed electric/electromagnetic field delivery with a variety of advanced spectroscopy and microscopy systems might enable breakthroughs in several fields:

- physical chemistry and electrochemistry: providing insight into the mechanisms of action of an electric field on non-covalent interactions in proteins,
- spectroscopy: aligning dipolar proteins with an electric field enables enhanced signals in X-ray scattering for single-molecule imaging, microwave, THz, and IR spectroscopy,
- structural biology: an electric field represents a physical handle for protein unfolding,
- physical biochemistry: the controlled activation of proteins in solution or a gaseous phase to probe the protein’s stability under different conditions,
- biotechnology: controlling the enzymatic activity of proteins in bulk,
- bioelectromagnetics: understanding in broad terms how an electric field affects biological systems at the molecular level,
- biosensing and analytics: controlling mass transport in biosensor applications.

## Theory and computation of electron transfer in proteins

Electron transfer (ET) at heterogeneous bio/metallic interfaces is traditionally studied by electrochemical methods, for example, protein film voltammetry [167–170]. Redox-active biomolecules such as metalloproteins are adsorbed onto the electrode surfaces, where their charge-transfer properties are probed by measuring current-voltage responses [171, 172]. Besides fundamental studies focused on the electronic behavior of the biomolecules, many interesting applications exploiting the natural biocompatibility, high selectivity, and enzymatic activity of suitable redox proteins have been developed, including manufacturing accurate biosensors, fuel cells, or enzyme-based biocatalysts [173–177]. Motivated by the efficient ET capabilities of metalloproteins, these biomolecules have started to be also incorporated into vacuum-based nanoelectronics, where solid-state protein junctions are created between metal contacts to form devices such as bio-based transistors or memristors [178–181]. However, unexpected quantum phenomena emerged at such bio/metallic interfaces, which soon attracted the attention of the broader research community [182, 183].

In aqueous solutions, redox-active proteins are known to transfer charge by the so-called incoherent hopping mechanism, theoretically described by the Marcus theory [184, 185]. The electron is localized to a redox site, where it stays for a long enough time to allow the relaxation of the molecular environment to the perturbed electrostatic potential. The energy needed to overcome the free energy barriers separating the individual redox sites is provided by the fluctuating electrostatic fields arising from the thermal motions of the protein and nearby hydration layers. Therefore, the hopping mechanism is strongly temperature-dependent. However, when single-protein junctions began to be probed by scanning tunneling microscopy or its electrochemical variant [186–189], unexpectedly high electric currents were detected, exhibiting practically no dependence on temperature [190]. Surprisingly, these data indicated that electrons could coherently tunnel through the protein, which is a fundamentally different charge-transport mechanism not typical for soft biomatter. Even more puzzlingly, the redox activity of the proteins, necessary for ET in their native environments, was shown to not affect the protein conductance when incorporated into metallic solid-state junctions [191, 192].

Knowledge of the adsorption structure of a protein on electrode surfaces at atomistic and electronic resolutions is essential for understanding the ET at the interfaces [9, 193, 194]. However, such details are hardly obtainable by experimental measurements. Therefore, computer simulations are often utilized to elucidate the structural data and the transport mechanism. Classical MD based on empirical potentials is used to predict representative adsorption structures on model surfaces, where image-charge interactions at the highly polarizable metal surfaces or covalent interactions (i.e., chemisorption) must be treated with special care [195–202]. Adsorption of the proteins into the desired conformation on the surface is often controlled by chemical modifications (for example, by introducing a reactive group to the biomolecular structure by protein engineering methods or coating the surface with suitable linkers) and can be enhanced by the application of external fields [195, 203–205].

**Fig. 8:**
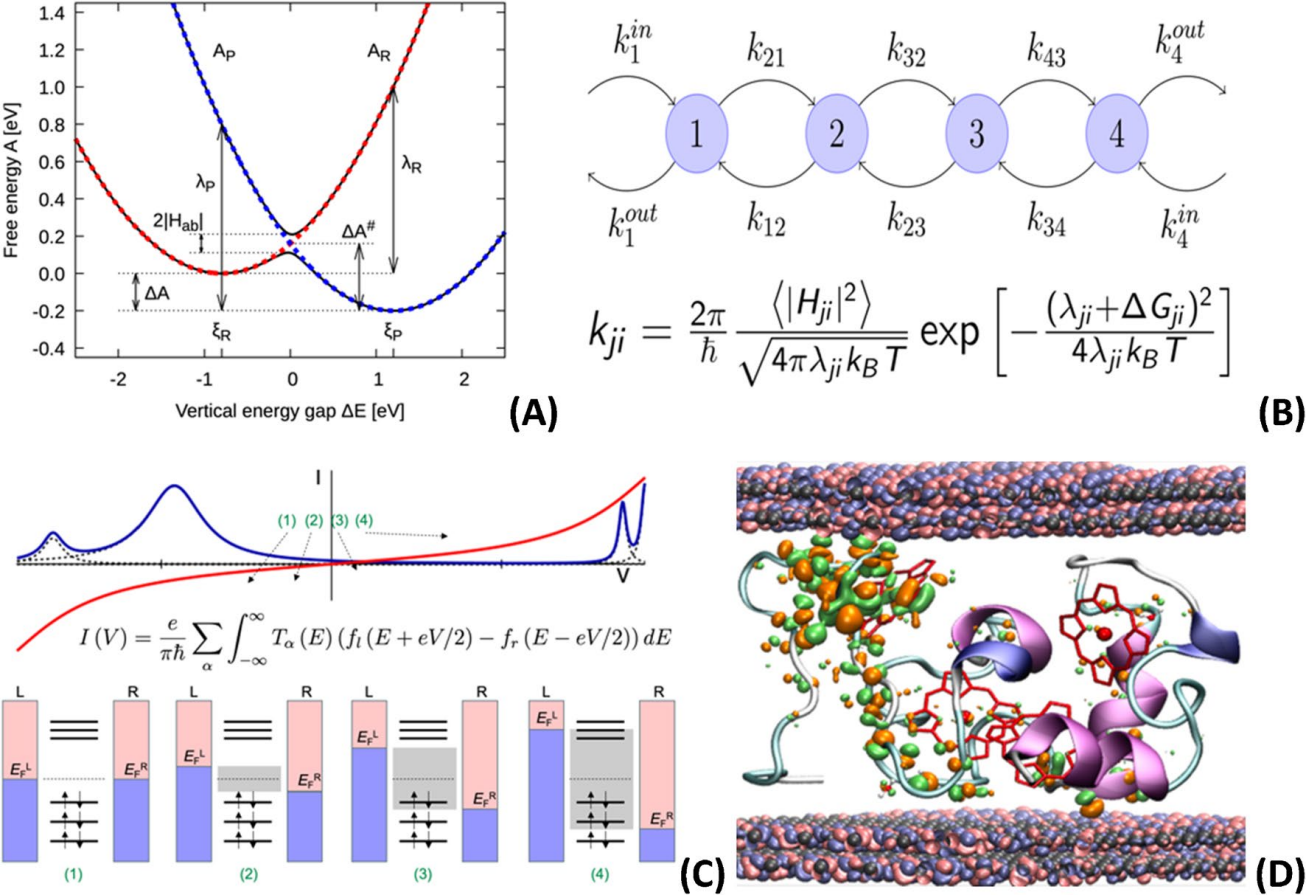
(**A**) Marcus parabolic free energy surfaces of initial (*A*_R_) and final (*A*_P_) charge states with indicated driving force (Δ*A*), reorganization free energies (λ), and electronic coupling element (*H*_ab_); (**B**) four-site hopping model and Marcus formula for electron transfer rate constant *k*_ij_; (**C**) schematic illustration of Landauer integration formula for tunneling current *I*(*V*); (**D**) small-tetraheme cytochrome junction between gold contacts with conduction channel shown as orange and green lobes

The electron localization typical for the hopping mechanism enables the application of combined quantum-mechanical/molecular mechanical (QM/MM) methods or their semi-empirical variants, such as the perturbed matrix method (PMM), where only the redox sites undergoing the oxidation/reduction processes are treated at the quantum level of theory, while the rest of the system is described by less-demanding empirical potentials [14, 206–208]. These methods are used to sample vertical ionization energy on the MD trajectories of oxidized/reduced systems, from which redox potentials and reorganization free energies are obtained by reconstructing Marcus parabolic free energy surfaces (Fig. 8A). The system response to the change in charge is often linear, which simplifies the calculation of these ET parameters [209–211]. The electronic coupling elements needed for determining the transfer rate constants can be obtained by different approaches, of which the most popular are the generalized Mulliken-Hush method [212, 213], fragment charge and energy difference techniques [214, 215], or DFT-based approaches such as FODFT [216, 217], CDFT [218–221], and POD [222–225]. For the interfacial ET between the electrode and the molecule, Marcus-Hush-Chidsey integrals have to be evaluated to obtain the rate constants [226, 227]. These are then brought together into a kinetic master equation for the site populations (Fig. 8B). By solving the equation, either by iterative techniques or the kinetic Monte Carlo method, the desired electronic flux is obtained [228–230].

Investigation of the coherent tunneling processes in protein junctions is, from the computational point of view, a much more challenging task. Transport at molecular junctions between metal contacts is usually studied within the framework of non-equilibrium Green’s function theory (NEGF) combined with tight-binding models or DFT [231–234]. Although this approach is formally accurate, in practice, it is only applicable to small systems because of its high computational demand. Studies of protein junctions are scarce because they require a full quantum description of relatively large proteins and electrode contacts. Such large-scale DFT calculations were recently demonstrated on the blue-copper protein azurin [235] and the small-tetraheme cytochrome [236, 237]. In the latter case, the tunneling currents were computed within the Landauer-Büttiker formalism (Fig. 8C), where the transmission function was treated in the Breit-Wigner approximation [238–240], regarding the transferred electrons as independent. Despite these necessary simplifications, the computed current-voltage curves agree well with experimental data, and the visualized conduction channels (Fig. 8D) helped with understanding the incoherent tunneling transport in such large molecules [236, 237].

Knowledge of the correct ET mechanism for the specific system is thus crucial for interpreting the measured data and performing computer simulations. While electron hopping is typical for electrochemical interfaces at one electrode, the tunneling mechanism can occur in protein junctions with two electrode contacts. The key factor controlling the ET mechanism in bio/metallic junctions is the electronic level alignment between the electrode and the molecule [182]. When there is a significant difference between the molecular redox states and the electrode Fermi level, the electron (hole) injection/ejection at the interface becomes the limiting step for the hopping mechanism, substantially lowering its efficiency, and the electronic charge is transported by coherent tunneling. For weakly adsorbed systems, which is usually the case with proteins, the alignment can be computationally estimated, for example, by the DFT+Σ technique, in which the Kohn-Sham DFT states obtained in generalized gradient approximation (GGA) are corrected for self-interaction error and missing image-charge interactions [241, 242]. These corrections can reach magnitudes of up to 1 eV and are essential for quantitative calculations of the interfacial ET [197]. However, as molecular electronics is a rapidly developing research field, new methods and approaches are being designed and optimized for these kinds of calculations.

Besides these fundamental concerns about the transport mechanism, the geometrical arrangements of the interfaces involving proteins and their electronic structures are affected by local EFs, as discussed in the previous section. These are dominated by their intrinsic components stemming from the structural and chemical composition, for example, by the location of various charged or polarized atomic groups [243–247]. The intrinsic fields are thus highly localized, oriented, and relatively strong. Therefore, they typically control the adsorption orientations and confinement [195, 248, 249]. On the other hand, the external fields, induced by applied bias potentials, are considerably weaker; however, they can vary over time. In computations, these fields are involved *via* a Lorentz force acting on the partial atomic charge in classical simulations or affecting the electronic density in quantum calculations, thus polarizing the affected structures [130]. While the structural field effects are usually studied by non-equilibrium MD techniques, NEGF can be utilized to capture electronic transport [231–234, 250]. Nevertheless, these approaches are computationally demanding and hardly applicable for large protein models, requiring more approximative treatments, as explained above.

The structure and functionalization of the electrode surfaces at nanoscales thus play a crucial role in protein electrochemistry. The electrode material, its surface facet orientation, and the surface dipole induced by reconstruction and termination processes determine the work function [251, 252] of the specific electrode and, as a result, the efficiency of the charge transfer mediated by the adsorbed biomolecules [182, 197, 237, 253]. Atomistic computer simulations can be employed to explore these effects and suggest key parameters for the desired adsorption and transport properties of specific systems. For example, suitable protein mutations with simultaneous surface functionalization by molecular linkers can be designed *in silico* to achieve the desired adsorption of enzymes onto biologically active surfaces [254, 255]. Furthermore, the incorporation of metallic nanoparticles at the surfaces has become popular in the last few decades due to their ability to induce locally enhanced fields. These techniques are often combined with plasma-polymeric surface coatings, ensuring good adsorption of both the nanoparticles as well as the proteins [256–259]. However, detailed knowledge of the atomistic details of these complex interfaces is required for further tuning and control of measurements and devices. Although such details are hard to obtain experimentally, they can be provided by computer simulations, which have proven to be useful tools for such applications.

## Conclusions and further prospects

Investigation of the intrinsic electroactivity of proteins is based on the reduction or oxidation of individual *aa* residues in their structure. These redox-active *aa* residues can be located on the surface of proteins, where they are fully accessible to the electrode surface. If another substance interacts with the surface of the protein, these *aa* residues can be modified (covalent bond) or blocked (non-covalent association), and the exchange of electrons between the protein and the electrode surface cannot take place. This can be clearly observed electrochemically at the surfaces of both mercury and carbon electrodes. Metal electrodes (such as gold or mercury) can also be used to investigate the oxidation or chemical modification of Cys residues, which is crucial to the function and structure of a whole range of proteins or peptides [18, 25]. In addition, a selective electrochemical method for analyzing His residues has recently been developed and demonstrated on various model peptides and proteins [23]. For these purposes, a mercury electrode was used, which is a very effective tool not only for the sensitive analysis of proteins but also for the analysis of their interactions and structural changes, such as aggregation, folding, or oxidative damage. In this sense, the monitoring of protein interactions and structural changes is based on electrochemically active *aa* residues inside the protein structure. These *aa* residues can be exposed to the surface of the electrode due to a structural (relaxation) change, e.g., unfolding. Today, the discontinuation of mercury electrodes in electrochemical research is a global trend that we perceive very negatively (for more details, see the literature [17, 260, 261]) since liquid mercury electrodes with an atomically smooth surface are excellent for evaluating and characterizing the electrochemical behavior of various compounds. Today, the electrochemistry of proteins and peptides is also increasingly connected with research on membrane systems and research on membrane proteins and their interactions [11, 15]. One of the important prerequisites in this research field is the fact that membranes can be anchored to electrode surfaces. Also, very often, the components of the lipid bilayer are not electrochemically active and thus do not directly interfere with the analysis of proteins that are reconstituted in these membranes. In our opinion, studies of the effect of the electric field on the structure and intermolecular interactions of proteins with other molecules or conductive (or biomimetic) surfaces are interesting research areas. In this review, we demonstrated this on cytoskeletal proteins using chip technologies and computational methods [133, 143, 155, 156, 160]. Intense short electrical pulses can modulate the network of non-covalent interactions of proteins and their components and thus interfere with their self-assembly processes, which can be utilized in protein molecular manipulation approaches.

In addition, we also point out the importance of computer simulations of processes associated with the structure of peptides and proteins immobilized on an electrically charged surface [190, 209, 210, 236]. Simultaneously, the simulation of electron transfer significantly helps to understand the biological function of redox-active proteins. In addition to ET, electrochemical research is also interested in proton transfer in proteins and research on other proton-dependent processes [262–264]. It would be beneficial to combine approaches based on the analysis of intrinsic electroactivity with approaches targeting ET at non-protein redox-active centers of the metalloproteins [91]. Also, the infrequent application of advanced computing techniques prevents an expansion of the interpretive framework of experimental studies. As for protein labeling, electrochemiluminescence approaches are considered to have a lot of potential [12, 13]. In general, the combination of optical (spectral) detection methods (*in situ* spectroelectrochemistry [265]), microscopic techniques, and electrochemistry (including electrochemical impedance spectroscopy [266]) has considerable potential for the future.

In this review, we have shown selected applications in protein electroanalysis. However, it is important to not only describe the advantages but also take into account the experimental difficulties and obstacles that can limit the application of electrochemistry in the research of protein interactions, both protein-low-molecular-weight-ligand interactions and protein-protein-DNA or -membrane interactions. In this sense, it is very important to understand the importance of adsorption effects and “protein surface denaturation” phenomena [267], which can lead to artifacts in interaction studies. At the same time, it is important to pay close attention to the influence of the electric field on the native structure of proteins for the correct interpretation of electrochemical data [18]. The above could help to orient oneself in the above-mentioned areas and, at the same time, see all the possibilities that electrochemistry offers for protein research and bioanalysis.

## Abbreviations

6-AQC: 6-aminoquinolyl-*N*-hydroxysuccinimidyl carbamate
*aa*: amino acid
AdT: adsorptive transfer
AuNPs: gold nanoparticles
CHER: catalytic hydrogen evolution reaction
CPS: chronopotentiometric stripping
CV: coefficient of variation
DFT: density functional theory
DME: dropping mercury electrode
DNA: deoxyribonucleic acid
DST: double-surface technique
ECL: electrochemiluminescence
EF: electric field
ET: electron transfer
GGA: generalized gradient approximation
HMDE: hanging mercury drop electrode
HSA: human serum albumin
LOD: limit of detection
MBA: 4-mercaptophenylboronic acid
MD: molecular dynamics
MGO: methylglyoxal
MIP: molecularly imprinted polymer
MT: microtubule
NDA: naphatalene-2,3-dicarboxyaldehyde
NEGF: non-equilibrium Green’s function theory
OPA: *o*-phthaldialdehyde
PEF: pulsed electric field
PMM: perturbed matrix method
PNP: protein nanopore
QM/MM: quantum-mechanical/molecular mechanical
RBL: rat basophilic leukemia
rHuEPO: recombinant human erythropoietin
RPE1: retinal pigment cells
SNP: solid nanopore
U2OS: human osteosarcoma cells
